# MTar: a computational microRNA target prediction architecture for human transcriptome

**DOI:** 10.1186/1471-2105-11-S1-S2

**Published:** 2010-01-18

**Authors:** Vinod Chandra, Reshmi Girijadevi, Achuthsankar S Nair, Sreenadhan S Pillai, Radhakrishna M Pillai

**Affiliations:** 1 Centre for Bioinformatics, University of Kerala, Thiruvananthapuram, India; 2 Department of Computer Applications, College of Engineering, Thiruvananthapuram, India; 3 Translational Cancer Research Laboratory, Rajiv Gandhi Centre for Biotechnology, Thiruvananthapuram, India; 4 Department of Instrumentation, N.S.S. College of Engineering, Pallakkad, , India

## Abstract

**Background:**

MicroRNAs (miRNAs) play an essential task in gene regulatory networks by inhibiting the expression of target mRNAs. As their mRNA targets are genes involved in important cell functions, there is a growing interest in identifying the relationship between miRNAs and their target mRNAs. So, there is now a imperative need to develop a computational method by which we can identify the target mRNAs of existing miRNAs. Here, we proposed an efficient machine learning model to unravel the relationship between miRNAs and their target mRNAs.

**Results:**

We present a novel computational architecture MTar for miRNA target prediction which reports 94.5% sensitivity and 90.5% specificity. We identified 16 positional, thermodynamic and structural parameters from the wet lab proven miRNA:mRNA pairs and MTar makes use of these parameters for miRNA target identification. It incorporates an Artificial Neural Network (ANN) verifier which is trained by wet lab proven microRNA targets. A number of hitherto unknown targets of many miRNA families were located using MTar. The method identifies all three potential miRNA targets (5' seed-only, 5' dominant, and 3' canonical) whereas the existing solutions focus on 5' complementarities alone.

**Conclusion:**

MTar, an ANN based architecture for identifying functional regulatory miRNA-mRNA interaction using predicted miRNA targets. The area of target prediction has received a new momentum with the function of a thermodynamic model incorporating target accessibility. This model incorporates sixteen structural, thermodynamic and positional features of residues in miRNA: mRNA pairs were employed to select target candidates. So our novel machine learning architecture, MTar is found to be more comprehensive than the existing methods in predicting miRNA targets, especially human transcritome.

## Background

MicroRNAs (miRNAs) are highly conserved, small but endogenous non-coding regulatory RNAs (18 to 24 nucleotides in length), that regulate gene expression. MicroRNAs can interact with target mRNAs, at specific sites either to induce cleavage of the message or to inhibit translation. Identifying their target mRNAs is vital in understanding cell functions like cell proliferation, differentiation and cell cycle. Also it throws light into the causes of diseases like lymphoma, leukemia, cancers and many cardiac problems where miRNA:mRNA pairing is found to play crucial roles [1,2].

MicroRNA is first transcribed as longer RNA molecule called pri-miRNA. The pri-miRNA is processed in the nucleus itself into hairpin RNA of 60 to 120 nucleotides by a protein complex consisting of the ribonuclease *Drosha *and an RNA binding protein *Pasha *[3,4]. This hairpin RNA, known as pre-miRNA, is transported to the cytoplasm via *exportin-5 *dependent mechanism. It is digested there by a dsRNA specific ribonuclease called *Dicer *[5] to form the mature miRNA. Mature miRNA is bounded by a complex, similar to the RNA induced silencing complex (RISC) that participates in RNA interface (RNAi) [6,7]. The mature miRNA makes base pairing with mRNA where complementarities exist between them. This results in target degradation in plants and destabilization in animals. In general, miRNAs can regulate gene expression either by translational inhibition or by mRNA destabilization.

**Figure 1:**
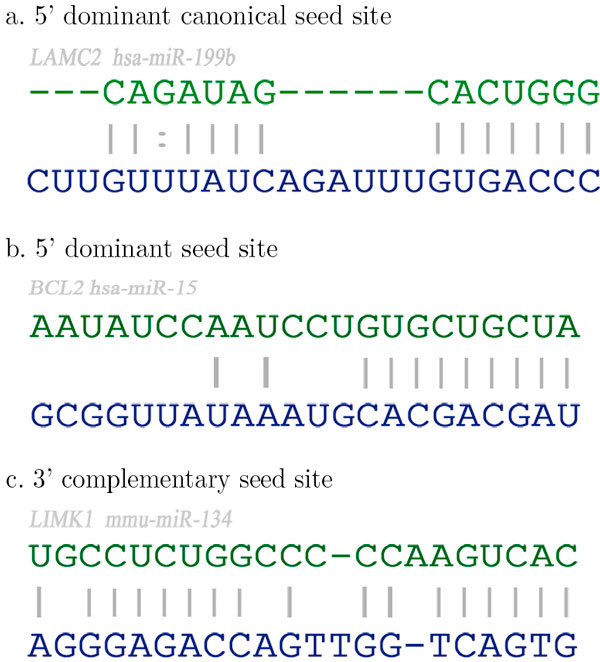
**Three type of targets**. Top lines are targets and bottom lines are miRNAs. (a) 5' dominant canonical seed site-miRNA (hsa-mir-199b) and mRNA (LAMC2). (b) 5' dominant seed-only site for miRNA (hsa-mir-15) and mRNA (BCL2). (c) 3' complementary seed site for mirRNA (mmu-miR-134) and mRNA (LIMK1).

The way microRNA and their targets interact in animals and plants is different in certain aspects. The plant miRNA exhibits perfect or nearly perfect base pairing with the target but in the case of animals, the pairing is rather imperfect. This makes the microRNA target identification problem in animals more complex compared to that in plants. Also miRNAs in plants bind to their targets within coding regions cleaving at single sites whereas most of the miRNA binding sites in animals are in the 3' un-translated regions (UTR) [8,9]. In animals, functional duplexes are found to be more variable in structure and they contain only short complementary sequence stretches, interrupted by gaps and mismatches. In animal miRNA:mRNA interactions, multiplicity (one miRNA targeting more than one gene) and cooperation (one gene targeted by several miRNAs) are very common but rare in the case of plants [10-12]. All these make the approaches in miRNA target prediction in plants and animals different in details [13,14]. We focused on the more complex animal (especially human) miRNA target identification problem while designing MTar. Experimental evidences show that the target needs enough complementarities in either the 3' end or in the 5' end of the miRNA for its binding. Based on these complementarities of miRNA: target duplex, the target sites can be divided into three main classes [15]. They are the 5' dominant seed site targets (5' seed-only), the 5' dominant canonical seed site targets (5' dominant) and the 3' complementary seed site targets (3' canonical). The 5' dominant canonical targets possess high complementarities in 5' end and a few complementary pairs in 3' end. The 5' dominant seed-only targets possess high complementarities in 5' end (of the miRNA) and only a very few or no complementary pairs in 3' end [16-18]. The seed-only sites have a perfect base pairing to the seed portion of 5' end of the miRNA and limited base pairing to 3' end of the miRNA. The 3' complimentary targets have high complementarities in 3' end and insufficient pairings in 5' end. The seed region of the miRNA is a consecutive stretch of seven or eight nucleotides at 5' end. The 3' complementary sites have an extensive base pairing to 3' end of the miRNA that compensate for imperfection or a shorter stretch of base pairing to a seed portion of the miRNA [15,19]. All of these site types are used to mediate regulation by miRNAs and show that the 3' complimentary class of target site is used to discriminate among individual members of miRNA families in vivo. A genome-wide statistical analysis shows that on an average one miRNA has approximately 100 evolutionarily conserved target sites, indicating that miRNAs regulate a large fraction of protein-coding genes. The three types of targets are shown in Figure 1.

Most of the existing solutions search for the complementarities of miRNA, only at the 3' end of mRNA thereby overlooking the complementarities in the 5' end of miRNA which is present in the third case (Figure 1c). As the pairing at 3' end of mRNA with 5' of miRNA is found to be more in number compared to the other, this results in missing of targets. We have paid adequate attention to this fact while designing MTar.

### Survey of existing solutions

A number of computational tools are available for animal and plant miRNA target identification. Of these, only MiRanda and RNAhybrid provide source code. Most of these approaches are based on evolutionary conservation and the presence of miRNA target sites in 3' UTRs of target mRNAs and their relatively better complementarities to 5' end of miRNAs. At the initial stages of microRNA target identification, researchers used near-perfect complementarities to predict miRNA targets for model species from plants. Tools like miRCheck [20], findmiRNA [21], PatScan [9] and mirU [22] are used for rapid prediction of miRNA targets in plants where perfect complementarities of miRNA and mRNA make the task easier. Though the targets for plant miRNAs can be identified on a genome-wide scale by searching for the ones that require a high degree of sequence complementarities, this cannot be used as such to find targets for animal miRNAs. The animal miRNAs tested till date pair imperfectly with their targets and act to control translation. Also the systematic analysis of the complete miRNA complement has confirmed the absence of targets with perfect or near-perfect sequence complementarities. So, target prediction in animal transcriptomes calls for more complex algorithms due to the imperfect complementarities of miRNA: mRNA pairs.

PicTar [23-25] predict miRNA targets in Drosophila and other species based on complementarities between miRNA and 3' UTR of mRNA sequence. PicTar used techniques like seed match, free energy calculation and species conservation. Its false positive rate has been estimated to be 30.0%.

TargetScan [26] is a tool used to predict miRNAs which bind to 3' UTRs of vertebrate transcriptomes. TargetScan could predict more than 451 human microRNA targets. TargetSanS [10], a modified version of TargetScan, omits multiple sites in each target and further filters the targets using thermodynamic stability criterion. Using this modified method more than 5300 human genes were predicted as possible targets of miRNAs [10]. The false positive rate varies between 22% to 31%.

MiRanda [12,27,28], a target prediction tool, relies on the evolutionary relationships between miRNAs and their targets. This tool focused to sequence matching of miRNA: mRNA pairs, by estimating energy of physical interaction. MiRanda was initially developed for predicting miRNA targets in Drosophila [27] and was later extended to find miRNA targets in mammals (human, mouse and rat) and Zebrafish [12]. The miRanda algorithm works by scanning for miRNA complementary pairs in the 3' UTR of an mRNA. Using this software, a large number of targets were identified including protein-coding genes in Homo sapiens. The false positive rate was estimated to be 24%.

The DIANA-microT [29] is a method based on the rules of single miRNA: mRNA pairing. It predicts targets which contain a single complementary site based on binding energies. MiTarget algorithm [30] combines thermodynamics based processing of RNA: RNA duplex interactions with the sequence analysis to predict miRNA targets. RNAhybrid is another computer program for predicting miRNA targets based on complementarities between miRNA and 3' UTR of coding sequence. This program was used to predict targets in Drosophila [11]. MovingTarget [31] is a program used to detect miRNA targets satisfying a set of biological constraints. Using this program more than 83 potential targets was predicted in Drosophila. MicroTar [32] is a program used to detect target sites in C.elegans, Drosophila and mouse by target complementarities and thermodynamic data. This algorithm uses predicted free energies of unbounded mRNA and putative mRNA:miRNA hetero dimmers, implicitly addressing the accessibility of the mRNA 3' UTR. This software is able to predict both conserved and non-conserved targets.

Anyway most of these existing tools make use of the complementarities in the 5' end of the miRNA alone. But MTar, the proposed computational method, can trap all the three types of targets (5' seed-only, 5' dominant and 3' canonical) and hence found to be more accurate. Multiplicity and cooperation which are common in animal miRNA: mRNA interactions are also handled effectively by MTar.

## Methods

### Training set

Experimentally verified microRNAs and their targets are required for training dataset preparation. In the data collection step, we excluded the sequences that were not verified by wet lab experiments in order to ensure the quality of the training data. We also excluded all the targets whose exact binding site could not be verified accurately. The dataset was selected based on two criteria. 1) The binding site of miRNA:target duplex should be known. 2) The target site sequences should match its corresponding references mRNA sequence in NCBI gene database.

The miRNAs were downloaded from miRBase database [33]. There are 706 reported human microRNA entries in the miRBase registry. The experimentally verified human microRNA targets were downloaded from Tarbase and miRecords registries. There are 609 human target sites for 443 genes by 107 miRNAs in Tarbase database [17]. The miRecords [34], contains 778 human records for 651 genes by 125 miRNAs. After filtered the target sites from these sources, the combined dataset consists of 882 human records for 741 genes by 138 miRNAs. The positive dataset needs three types of target classes (5' seed-only, 5' dominant, and 3' canonical). The classification was done based on the complementarity in the seed region. Seed region is the region of the nucleotides from 2-8 or 2-9 positions of the miRNA from the 5' end.

Randomly generated negative examples were not included in the training set, as such sequences are often found to interact with miRNAs due to their low signal to noise ratios as it is evident from previous studies [15,26,29]. Deletion of target positions on the target miRNA sequence can give a large number of negative examples. We generate a common negative dataset contains non miRNA:mRNA target sites with different parameter score. We collected examples with more than 4 mer matched at their seed part. Alignments of sequences in the training datasets were thoroughly checked in order to avoid ambiguities. The training set consisted of 350 examples with 150 positives and 200 negatives. The selection of positive dataset was based on the availability of experimentally verified target sites for the three class of targets in Tarbase registry. From these sequences three separate training datasets were created for three target classes (5' seed-only, 5' dominant, and 3' canonical). The training dataset contains 40 positives and 56 negatives for 3' canonical target class, 58 positives and 74 negatives for 5' dominant target class and 52 positives and 70 negatives for 5' seed-only target class.

### Parameter selection

Analysis of experimentally verified miRNA target sites give a number of parameter features [13,15,29,35-38]. We investigate the role of each parameters in miRNA:miRNA formation, to select 16 most relevant parameters which are tabulated in Table 1. These parameters are classified into three categories. They are, structural (numbered 1 through 8), thermodynamic (numbered 9 through 12) and positional (numbered 13 through 16) features of the miRNAtarget sites. The parameter and their value calculation are given in Table 1. For the structural and thermodynamic features, we divided the secondary structure alignment into three parts - 5' part (seed part), 3' part and total alignment. The thermodynamic properties like free energy and hybridization energy were calculated using RNAfold and RNAcofold [39]. The thermodynamic features are very effective in the case of short matches identification in miRNA:mRNA pair [11]. The structural parameters are positive or negative real numbers. The position based features of miRNA:mRNA is important because of their shaping mechanism in the seed region [15,40]. Positional parameters are integers. Each position amounts to any one of the four values depending upon whether it is G:C match or A:U match or G:U match or a mismatch.

**Table 1:** Parameters used for miRNA target prediction

No	Parameter	Parameter Description
1	Seed score	Obtained by the sum of pair scores in the seed region. G:C and A:U with 5, G:U with 2 and the others with -3
2	Out seed score	Obtained by the sum of pair scores in a non-seed region. G:C and A:U with 5, G:U with 2 and the others with -3
3	WC pairs	Number of WC pairs in the duplex
4	Wobble pairs	Number of wobble pairs in the duplex
5	Mismatches	Number of mismatches in duplex
6	Length-bulge	Length of largest bulge in the duplex
7	Number-bulges	Number of bulges in the duplex
8	Proportion	Proportion of 'A', 'G','C' and 'U' in the target sequence
9	Free energy	Free energy is calculated using RNAfold of the target sequence
10	Hybridization Energy	Calculated using RNAfold for a duplex formed by the miRNA and its target. RNAfold is the part of ViennaRNA package [39]
11	Normalized free energy	NFE = (-1 * free energy of target sequence)/log(length of target * length of miRNA)
12	Difference in hybridization energy	Difference in the hybridization energies of miRNA:perfect target (reverse complement of miRNA) duplex and miRNA: target duplex
13	Positional pair score	This score is considered by both pairing and positioning. G:C and A:U with 5, G:U with 1, all other mismatches awarded with -3 and the mismatches containing gaps are awarded with -1. Positional weight is 1 for all non-seed position and 2 for all seed position. The total score is obtained by the sum of the product of the weight and the corresponding pair score through out the mirna: target duplex
14	Matrix score	Obtained by the sum of diagonal elements in the matrix formed by miRNA and its target. The scores are based on WC pairs: 5, Wobble pairs: 2, Inserts: -1, Deletes: -1, Symmetric mismatches: -3, Mismatches: -2
15	Deviation matrix score	Deviation of the matrix score with the score obtained by a perfect target ie, the reverse complement of miRNA
16	Deviation positional score	Deviation of the positional pair score with the score obtained with a perfect target

### The method

**Figure 2:**
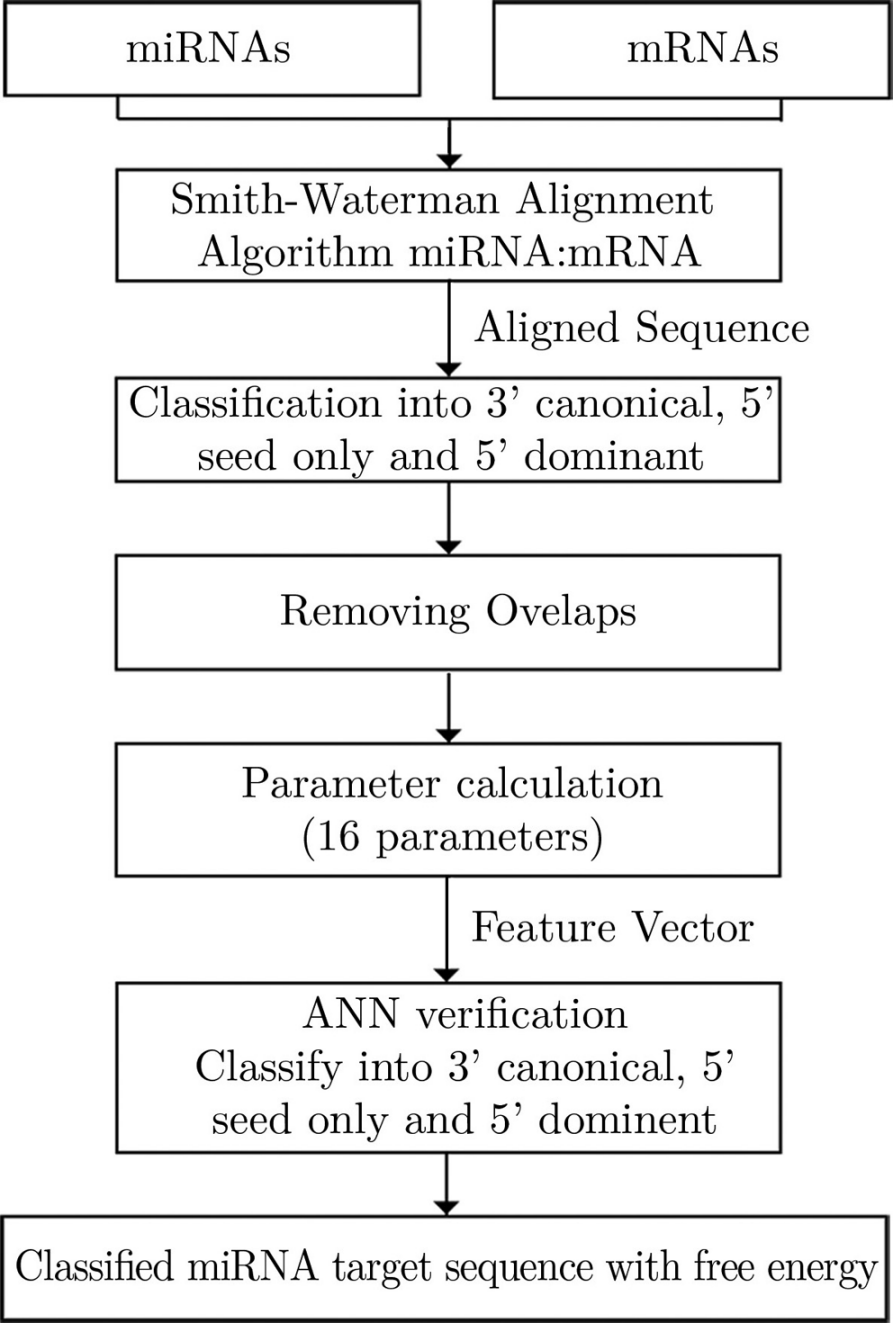
**Structure of MTar**.

Figure 2 summarizes the computational structure of MTar. To search for all possible alignments in each miRNA:mRNA pairs, segment of mRNA with length equal to the length of miRNA plus 10 nucleotide, starting from the first position, are selected. To locate miRNA targets, the miRNA sequence input is first aligned with the given mRNA target sequence using modified Smith-Waterman local alignment algorithm [41]. In the algorithm, gaps are allowed between the miRNA: mRNA pairs, but mismatches are preferred to gaps by giving a higher penalty for gaps. A scoring scheme in which each Watson-Crick pair (G:C and A:U) enjoys a score of 5, each G:U pair , a score of 1 and all others a score of -3, is employed. The gap opening amounts to -8 and a gap extension is less penalized with a score of -2. Based on this rule, a score S is computed for an alignment.

Next step is to classify the three types of target candidates. For this, the aligned sequences are first checked for seed complementarity. If selected region of the miRNA:mRNA pair has a seed complementarity, then the remaining region of the pair undergoes the out-seed complementarity. To calculate a seed complementarity score, the Watson-Crick (WC) pairs get higher priority than that of the wobble pairs. We calculate a seed complementarity score for G:C matches, A:U matches, G:U matches and other mismatches, for a sequence pair. The complementarity score in the seed region and out seed region are used to classify the three types of targets. The complementarity score calculation for the three different classes are:

1. *5' seed-only*: For this class, a minimum of 6 WC pairs and no mismatch in the seed region are allowed. No G:U pair is allowed in the seed region. The non-seed region may contain a minimum of 4 matching pairs including G:U pairs.

2. *5' dominant*: A minimum of 5 WC pairs, with one mismatch and a maximum of 2 G:U pairs are allowed in the seed region. Minimum 5 matching pairs including G:U pairs should be in the non-seed region.

3. *3' Canonical*: A minimum of 3 WC pairs, 4 mismatches and maximum 3 G:U pairs are allowed in the seed region. The non-seed region should contain a minimum 7 matching pairs including G:U pairs.

If the selected region does not belong to any of these classes, it is not considered for further processing. The predictions are strictly based on the class of the targets. Each of these segments is aligned with the miRNA to locate potential target candidates belonging to all the three different categories (5' seed-only, 5' dominant and 3' canonical) based on the nature of complementarities. After this, all overlaps are removed by filtering. Then, using the parameters listed in Table 1, a feature vector is formed by giving appropriate weights to the parameters for each of the candidates. Then they are submitted to an ANN classifier. The threshold levels for the parameters are different for targets of different categories. The validated potential targets are displayed along with their class to which they belong.

### ANN classifier

In this study, artificial neural networks (ANNs) were chosen as the tool for miRNA target verification as they are powerful classifiers whose ability to cope with complex data and their potential for modeling data of high non-linearity [42,43]. We used a feed forward three layer multi layer perceptron (MLP) for the classification of target sites. Calculated value of the parameters (Table 1) of miRNA:mRNA pair was selected as a 16 dimensional vector and fed into 16 input nodes. Output is set as either '1', if the output pattern is true and -1', if the output pattern is false. Hence there is one unit in the output layer of the ANN. The number of units in the hidden layer was chosen as nine, by trail and error. We used a sigmoid transfer function as the activation function. Back propagation algorithm was used to train the network. The training can be performed with use of several optimization schemes and there is access to exact partial derivatives of network outputs versus its inputs. The over fitting was avoided and this package makes automatic normalization of input data [34]. The learning rate and momentum were initially set at 0.2 and 0.8 respectively.

The training dataset is divided into two subsets. First subset (70% of the total training data) were used to train the neural network. Second subset was used to stop the training process once the model had reached the performance conditions like optimal error value thus preventing over training. Once the training is stopped, the efficiency of the model was further assessed by presenting another data subset, to determine the performance for unseen cases which were not involved in the training process. Optimization was done by repeating the process with different data subsets. The optimization needs nearly 500 epochs for this network. Three separate ANNs for each target class (5' seed-only, 5' dominant and 3' canonical) were trained to validate the target candidates of three different classes. Each ANN was trained with their training set and optimized the network as discussed above.

## Results and discussion

### Performance evaluation

Extensive evaluation of MTar architecture was carried out using human genome. We could computationally predict 2663 target sites including 819 experimentally verified targets of 129 miRNAs (MFE below 17.0 Kcal/mol). For evaluation, the miRNA test data was downloaded from miRBase registry and the mRNA sequences from NCBI RefSeq and Biomart [44] cites.

**Figure 3:**
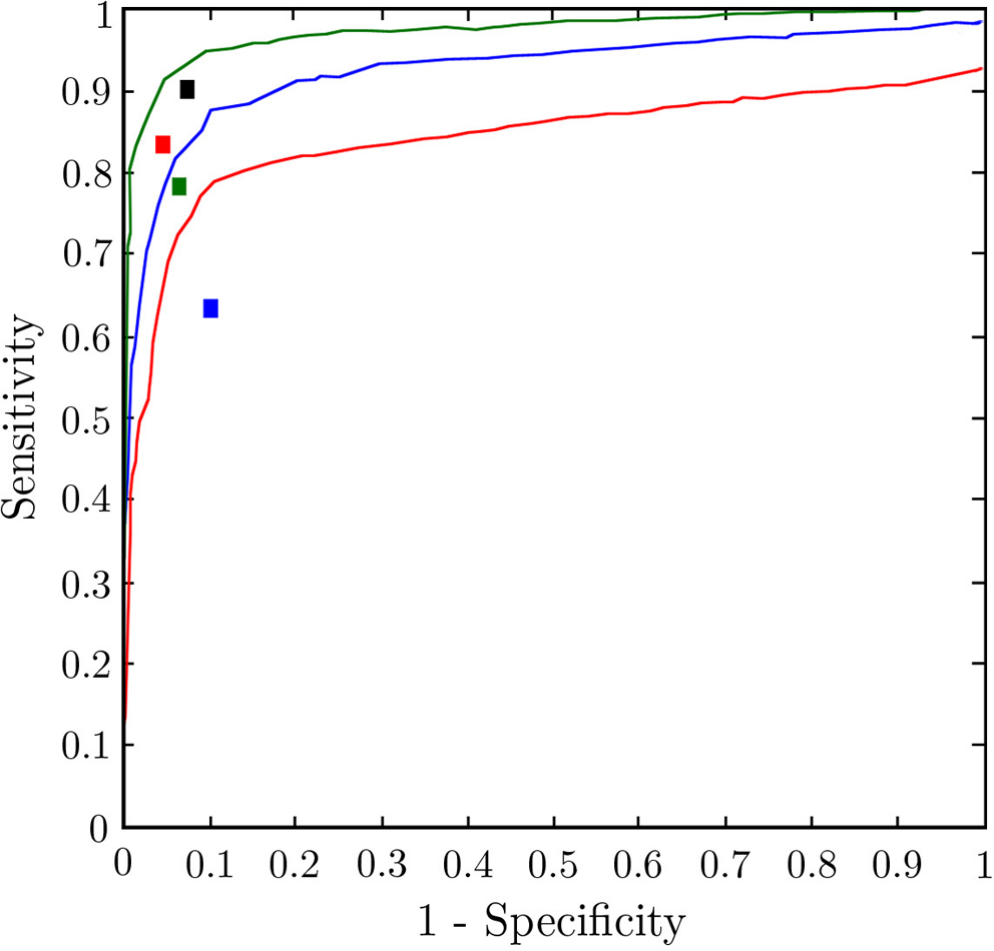
**ROC curve of MTar along with a comparison of features**. (a) Combined three properties (blue curve). (b) Positional and thermodynamic properties (green curve). (c) Structural and thermodynamic properties (light blue curve).(d) Structural and Positional properties (red curve). The rectangle denotes performance of the other tools. MiTarget (Black mark), Miranda (Green mark) and Target-Scan (Red mark). True positive rate (sensitivity) on Y-axis and false positive rate (1 - specificity) on X-axis.

We analyzed the performance of MTar using Receiver Operator Characteristic (ROC) curve which is shown in Figure 3. ROC is a plot of the true positive rate (sensitivity) against the false positive rate (1-specificity) for the different possible cutoffs of a diagnostic test. The test dataset contains experimentally verified target sites from Tarbase and miRecords databases. We confirmed that, any of the test data was not included in the training dataset. There are 190 positives and 200 negative examples selected in the test dataset. First, we tested MTar with all three features (positional, thermodynamic and structural features) combined. The area under the ROC curve was found to be 96%. The performance of MTar in terms of sensitivity (94.5%) and specificity (90.5%) is obvious. The threshold cutoff of MTar is 0.98 at this point. Then we investigated the effect of combining two features. For that, we tested MTar by taking only positional and thermodynamic properties at a time. The ROC area was decreased by 9.6%. Next we combined structural and thermodynamic properties together and then the ROC area decreased by 12.6%. Then we combined structural and positional properties together and tested MTar by the same dataset. The ROC area was decreased by 11.2%. From these experiments we could establish that all the three features (positional, thermodynamic and structural features) should be taken into account for improved miRNA target identification.

### Role of parameter in target prediction

**Figure 4:**
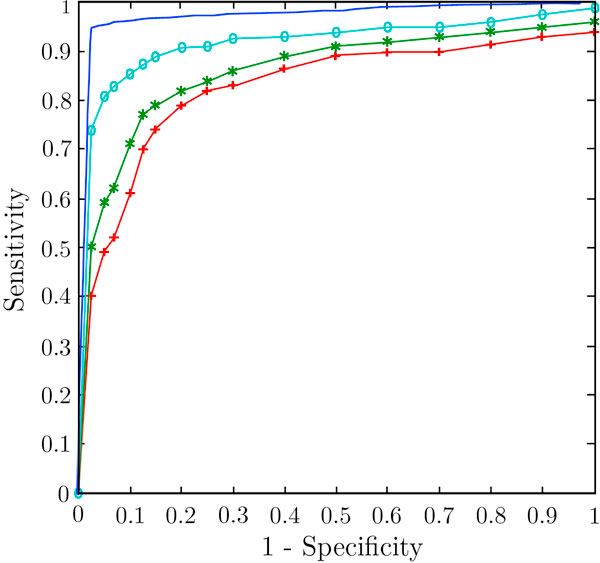
**Performance of MTar with respect to the features selected**. Plus symbol (+) line for top three features (red curve), asterisk symbol (*) line for top seven features (green curve), circle marked line for top 12 features (light blue curve) and top plane curve for complete feature set (blue curve).

We also investigated the role of various parameters in miRNA target prediction. For ranking the features, we used Weka software [45]. This software is a machine learning algorithm based data mining software used for classification and visualization of dataset. Using a dataset, the features are ranked and are shown in Table 2. Positional features got high ranks compared to structural and thermodynamic parameters. We investigated the performance of MTar with different combinations of various parameters. The same training and testing dataset (used for ROC curve analysis) was used for analyzing top 3, 7, 12 and 16 features respectively (Table 1). The ROC curve for each test is shown in Figure 4. The prediction tool gave its best performance when all the features were included in dataset. Specificity and sensitivity were also significantly increased when the training was done with all the features given in Table 1.

**Table 2:** Ranked parameters

Rank	Score	Parameter
1	84.3	Seed score
2	83.4	Positional pair score
3	81.0	Matrix score
4	80.6	WC pairs
5	78.8	Deviation positional score
6	77.2	Deviation matrix score
7	75.0	Out seed score
8	72.5	Wobble pairs
9	70.4	Normalized free energy
10	68.6	Mismatches
11	68.3	Hybridization energy
12	68.0	Free energy
13	65.5	Difference in hybridization energy
14	57.0	Length - bulge
15	52.4	Number-bulges
16	50.1	Proportion

### Comparison with existing approaches

A comparison of the summary of exhaustive runs of MTar and other existing solutions can be found in Table 3 and Table 4. Table 3 tabulates the number of experimentally verified targets, for all 138 experimentally verified miRNAs in human genome using MTar and five other existing solutions. Table 4 furnishes the total number of targets (Computationally predicted including experimentally verified) predicted by the same tools for all 138 experimentally verified miRNAs. From the tables the specificity of our tool is high as the other tools, because the other tools predict more false positives. These tables clearly point out the merits of MTar. MTar's superiority compared to other programs is obvious from Table 3 and Table 4.

**Table 3:** miRNA:mRNA targets result comparison (882 target sites for 138 miRNAs and 741 mRNAs)

Tool used	MTar	MiRanda	TargetScan	RNA22	PicTar	MiTarget
No. of targets predicted	819	670	689	287	466	767

**Table 4:** Total targets predicted by each tool for the experimentally proven miRNAs

Tool	No of microRNAs	Total Targets
MTar	129	2663
MiRanda	124	333809
TargetScan	89	132804
RNA22	105	29770
PicTar	107	63812
MiTarget	121	236109

The MiTarget is one of the latest web based miRNA target prediction tool, which predict most of the 5' seed-only and 5' dominant miRNA target regions but it fails in identifying 3' canonical targets. This may be the due to picking the features from 5' part of the miRNA only while it is binding to the 3' part of the mRNA and ignoring the 3' part of the miRNA target.

After testing the MTar with a set of data, the test was repeated for MiRanda, TargetScan, RNA22, PicTar and MiTarget, with the same dataset. Figure 3 depicts the receiver operator characteristics curve for MTar along with a comparison of other three tools (MiTarget, MiRanda and TargetScan). The other three methods not provide cutoffs, so ROC generation was difficult. MiRanda shows a specificity of 82% by the test dataset. The specificity of PicTar nearly 70% and that of TargetScan is comes around 80% by the same dataset. We are not ploted the position of these two tools in Figure 3, due to their low specificity. The sensitivity of these tools are seen in Table 3. MTar gives an average accuracy of 92.8%, sensitivity as high as 94.5% and a specificity of 90.5% for the miRNA targets in the testing dataset.

The relative merit and comprehensiveness of MTar can be attributed to the following facts. First, the approach identifies all the three types of targets (5' seed-only, 5' dominant, and 3' canonical) in a single framework unlike other approaches. The second reason is the use of all the three properties (positional, thermodynamic and structural features) of the miRNA:mRNA duplex for the target identification. Third, multiple target sites are treated differently by set of user-defined biological constraints. A user can fix the free energy cutoff criteria of a miRNA:mRNA pair. Finally, our method is a desktop application for human transcriptome and also extensible to the other species.

**Figure 5:**
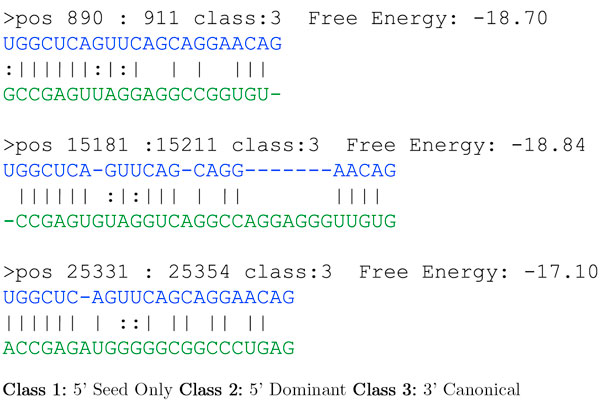
**Sample output of MTar for an input sequence**. miRNA (hsa-miR-24) and mRNA (ENSG00000148400, Chromosome:NCBI36:9:138508117:138560735:-1). In the alignment miRNA (blue colour) and mRNA duplex (green colour) are shown. Class 1 : 5' Seed-only (5' dominant canonical seed sites), Class 2 : 5' Dominant (5' dominant seed site) and Class 3 : 3' Canonical (3' complementary seed site).

A sample output of MTar with folding energy is shown in Figure 5. The user selected folding energy cutoff is below -17.0 Kcal/mol. The average MFE from the experimentally verified human miRNA target was calculated as -17.4 Kcal/mol.

## Conclusion

In this paper, we proposed a novel computational method for microRNA target prediction (MTar), which can identify all known three types of miRNA targets (5' seed-only, 5' dominant, and 3' canonical). Most of the computational methods for miRNA target prediction combine 5' seed matches, thermodynamic stability and conservation analysis in order to maximize specificity of the algorithms. Especially, evolutionary conservation is found to be an excellent tool for filtering out false positives thereby increasing specificity. MTar uses all these features and also takes into consideration the structural and positional features of miRNA: mRNA formation. The method makes use of three ANN verifiers, thoroughly trained by proved biological data. Sixteen positional, thermodynamic and structural features of miRNA: mRNA pairs were employed to select target candidates. Extensive evaluation of the proposed method was carried out using human genome. MTar identifies potential targets of 101 experimentally proved microRNAs. The performance of MTar was compared against existing solutions and the method is found to be more accurate. Our method predicts the three types of targets with a prominent accuracy (92.8%), sensitivity (94.5%) and specificity (90.5%). The false positive rate of MTar is 9.5% for MFE ≤ -17.0 Kcal/mol. The false positive rate can still be reduced by adjusting the MFE between miRNA:mRNA pairs but at the cost of lowered sensitivity. MTar has another edge due to its trainability as the performance can still be improved.

## Competing interests

The authors declare that they have no competing interests.

## Authors' contributions

VC conceived the research, carried out prediction and analysis of the miRNA. VC designed script and code to analyze data. VC and ASN performed the experiments and drafted the manuscript. SS and RG helped to analyze the data and manuscript preparation. MRP conceived the experiments. All authors read and approved the final manuscript.
